# Cardiac optical mapping – State-of-the-art and future challenges

**DOI:** 10.1016/j.biocel.2020.105804

**Published:** 2020-09

**Authors:** Christopher O’Shea, S. Nashitha Kabir, Andrew P. Holmes, Ming Lei, Larissa Fabritz, Kashif Rajpoot, Davor Pavlovic

**Affiliations:** a Institute of Cardiovascular Sciences, University of Birmingham, Birmingham, UK; b Institute of Clinical Sciences, University of Birmingham, Birmingham, UK; c Department of Pharmacology, University of Oxford, Oxford, UK; d Department Cardiology, University Hospital Birmingham, Birmingham, UK; e School of Computer Science, University of Birmingham, Birmingham, UK

**Keywords:** Cardiac optical mapping, Fluorescent imaging, Electrophysiology, Arrhythmia, Action potential, Calcium transient

## Abstract

Cardiac optical mapping utilises fluorescent dyes to directly image the electrical function of the heart at a high spatio-temporal resolution which far exceeds electrode techniques. It has therefore become an invaluable tool in cardiac electrophysiological research to map the propagation of heterogeneous electrical signals across the myocardium. In this review, we introduce the principles behind cardiac optical mapping and discuss some of the challenges and state of the art in the field. Key advancements discussed include newly developed fluorescent indicators, tools for the analysis of complex datasets, panoramic imaging systems and technical and computational approaches to realise optical mapping in freely beating hearts.

## Highlights

• Cardiac optical mapping is a fluorescent imaging method to study electrical behaviour and calcium handling in the heart.
• Optical mapping provides higher spatio-temporal resolution than electrode techniques, allowing unique insights into cardiac electrophysiology in health and disease from a variety of pre-clinical models.
• Both transmembrane voltage and intracellular calcium dynamics can be studied with the use of appropriate fluorescent dyes.
• Optical mapping has traditionally required the use of mechanical uncouplers, however computational and technical developments have lessened the requirement for these agents.
• Novel fluorescent dyes have been developed to optimise spectral properties, experimental timescales, biological compatibility and fluorescence output.
• The combination of these developments has made possible novel mapping experiments, including recent *in vivo* application of the technique.

## 1 Introduction

The transmission of electrical impulses in the heart is vital for the coordinated contraction of the myocardium. Disturbances in how these electrical signals, namely the cardiac action potential, are generated or propagated can cause disruptions to maintenance of normal heart rhythm or rate. These disturbances are typically known as cardiac arrhythmias. Cardiac arrhythmias, and cardiovascular disease in general, present a substantial health burden. Arrhythmias can lead to sudden death, stroke or heart failure (Benjamin et al., 2019). Arrhythmogenic triggers in complex conditions such as atrial fibrillation and heart failure remain poorly understood. It is therefore vital to develop tools to better understand healthy and abnormal cardiac electrical function to inform more effective treatment for cardiac arrhythmias.

Since its introduction over 40 years ago, optical mapping has transformed pre-clinical cardiac electrophysiology. Utilising fluorescent indicators and high-speed cameras, electrical activation, repolarisation and calcium handling can be imaged across myocardial preparations at spatial resolutions far surpassing electrode techniques. Since the first recording of optical action potentials (OAPs) in 1976 (Salama and Morad, 1976), optical mapping has been applied to whole hearts, isolated atria, wedge preparations, cardiac slices and cellular monolayers (O'Shea et al., 2019c).

The unique characteristics of optical mapping has advanced our understanding of electrical function and dysfunction in the heart. The hypotheses of spiral waves in atrial fibrillation (Jalife, 2003), the creation and consequence of ‘virtual electrodes’ in defibrillation (Ripplinger et al., 2009), and cardiac transmural heterogeneity (Wen et al., 2018) have all been elucidated using optical mapping technology.

These insights and the continued uptake of optical mapping in cardiac research has been fuelled by significant technical advances in camera technology, fluorescent dyes and experimental design. In this review we provide a brief introduction to the basic principles and tools underpinning optical mapping. We then focus on the current state-of-the-art and challenges.

## 2 Basics of optical mapping

**Fig. 1:**
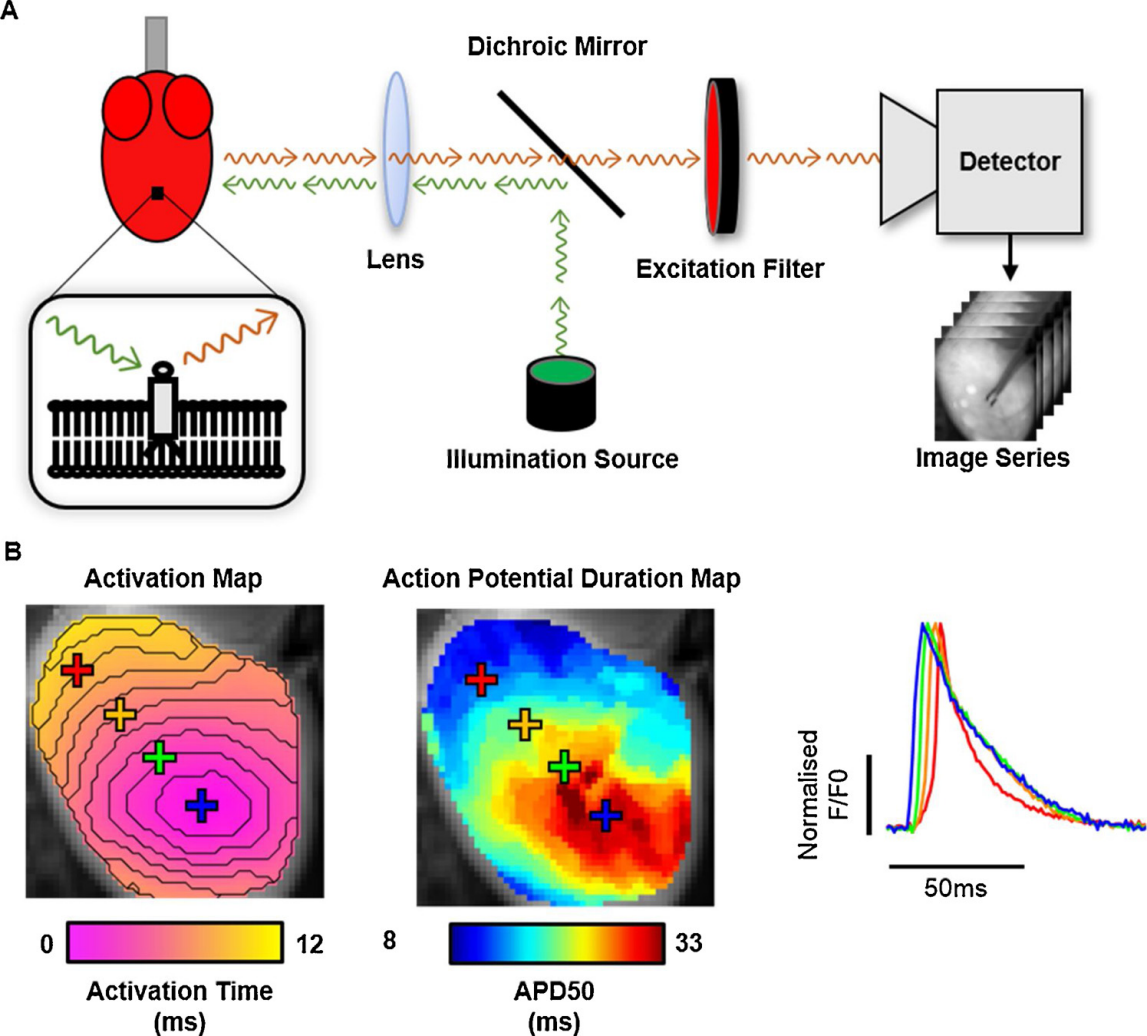
**Cardiac optical mapping setup, data and analysis.** A) Schematic representation of a typical optical mapping setup for imaging a potentiometric dye loaded cardiac preparation (left). Inset shows the fluorescent indicator embedded within the cellular membrane. The potentiometric dye is excited by photons (green arrows) from an illumination source. This causes the release of fluorescent photons (red arrows) whose spectral properties depend on the transmembrane voltage. Fluorescent photons are filtered from the illumination photons and directed to a high-density imaging detector to produce a time series dataset. B) Example of analyses possible from optical mapping datasets, including activation and signal morphology mapping (left panel). Right panel shows examples of the raw signals produced at each pixel of an optical mapping dataset, in this case optical action potentials from mouse ventricles. Y axis denotes normalised fractional fluorescence change (F/F0) from baseline fluorescence level (F0). (For interpretation of the references to colour in this figure legend, the reader is referred to the web version of this article).

A basic optical mapping setup (Fig. 1a) can be split into 3 major components: i) cardiac preparation loaded with fluorescent dye, ii) an optical setup for dye excitation and collection of fluorescent output and iii) a detector for imaging. By successful implementation of these components, action potential (or calcium transient) propagation and morphology can be directly imaged at high spatio-temporal resolution, see Fig. 1b (O'Shea et al., 2019a).

In whole heart preparations, fluorescent dyes are commonly introduced *via* Langendorff perfusion of the coronary arteries. Superfusion of the atria, myocardial slices and wedges is also utilised by some investigators. Potentiometric dyes are the most widely used. These dyes are designed to embed within the cell membrane and have a fluorescent output that is responsive to the transmembrane voltage of the cell, allowing the recording of optical actions potentials. The use of these dyes however is not without limitations, see section 3. Optical mapping also utilises intracellular calcium indicators which exhibit increased fluorescent output on chelation of Ca^2+^ ions, allowing high speed imaging of optical calcium transients, and simultaneous recording of action potentials (O'Shea et al., 2019a).

Successful optical mapping relies on an appropriate optical and imaging setup (Fig. 1a). Firstly, an illumination source is required, ideally illuminating the entire sample homogenously. Due to low heat emission, narrow spectral profile and long lifetime, LED illumination is most used although Tungsten-Halogen lamps, Mercury/Xeon arc lamps and lasers are also utilised. The illumination photons are then directed on to the cardiac sample, where they excite the indicator and elicit release of fluorescent photons. Optical filtering is required to separate illumination and emission photons, and to effectively image the optical signals. Pioneering optical mapping experiments utilised photodiode arrays, however in current optical mapping setups CMOS and CCD cameras dominate (O'Shea et al., 2019a).

## 3 State-of-the-art and challenges

### 3.1 Fluorescent dyes

As outlined, optical mapping relies on the use of fluorescent dyes. Most commonly, these are potentiometric dyes, and in particular small molecule synthetic indicators such as the electrochromism based styryl dyes di-4-ANEPPS or rh-237. Although these molecules exhibit fast responsive fluorescent changes (femto- to picosecond) in response to transmembrane voltage, there are several important drawbacks to their use. The fractional changes in fluorescence output are relatively small (∼10 %), while photobleaching, phototoxicity and dye internalisation can limit experimental time scales and lead to non-physiological alterations in the cardiac preparation (Matiukas et al., 2007; Larsen et al., 2012).

Spectral characteristics of these dyes can also present challenges for use in cardiac tissue. The di-4-ANEPPS for example requires excitation by blue/green light. At these wavelengths, light is strongly absorbed by biological chromophores in blood and tissue. Therefore, signals can only be derived from near the preparation surface (preventing transmural analysis). Although isolated studies using di-4-ANEPPS have achieved optical mapping in blood perfused preparations (Zaitsev et al., 2003), crystalloid solutions are commonly required to achieve sufficient signal quality. Crystalloid solutions however are less able to meet the metabolic and oxygen demands of cardiac tissue, a problem that is compounded in freely beating preparations and can result in hypoxia and ischemia (Garrott et al., 2017).

Consequently, efforts to develop better fluorescent dyes for optical mapping experiments continue, including recent development of red-shifted dyes. By shifting excitation ranges to red wavelengths (*e.g.* 650 nm as excitation wavelength for di-4-ANBDQPQ and di-4-ANBDQBS), fractional changes in fluorescence can be achieved in blood perfused myocardium that far exceed those of di-4-ANEPPS in the same preparations (Matiukas et al., 2007). Penetration depth is increased due to lack of absorption of excitation photons, and these dyes have also been shown to be less resistant to signal decay by photobleaching and dye internalisation. These properties have allowed optical mapping of *in vivo* preparations (Lee et al., 2019), and transillumination approaches for transmural integration (Mitrea et al., 2011).

Recently, photo-induced electron transfer has been exploited to produce fast responsive dyes with higher fractional fluorescent changes, such as FluoVolt. FluoVolt has been used for two-photon excitation studies in mouse hearts, an alternative to transillumination as a method for transmural integration (Salerno et al., 2019). Furthermore, signal quality and phototoxic side effects can be alleviated using genetically encoded sensors (*e.g.* Quasar voltage sensors, GCaMP calcium sensor), which provide unique opportunity for combination with optogenetic techniques and chronic investigation (O'Shea et al., 2019a). However, genetic sensors exhibit slower response times when compared with synthetic dyes (Broyles et al., 2018). For a wider discussion on voltage and calcium indicators, the reader is directed to relevant reviews (Broyles et al., 2018; O'Shea et al., 2019a).

### 3.2 Processing and analysis of optical mapping data

**Fig. 2:**
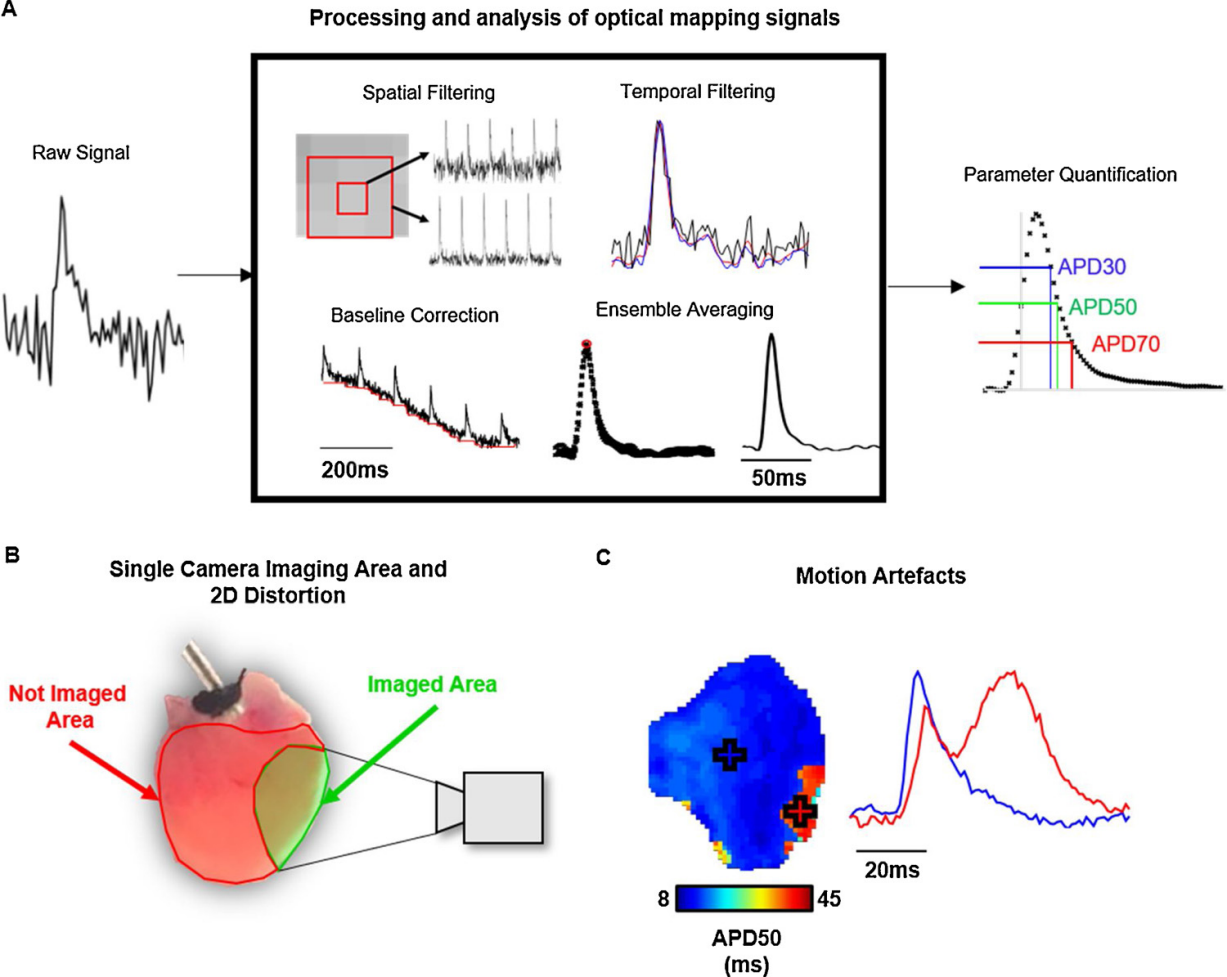
**Challenges in cardiac optical mapping.** A) Example of processing techniques that are often applied on all pixels in an optical mapping dataset to enhance the signal to noise ratio of the raw data (left) to allow effective parameter quantification (right). Note: The exact methods and sequence of processing methods used will vary depending on experimental setup, model and analysis software used. B) Schematic representation of a typical imaged area (green) from a mouse whole heart using a single camera setup. The red area shows the area of the epicardial ventricular surface that is not imaged. C) Example signals from a mouse atrium where motion artefacts are present. In the area with prolonged APD (red), contraction has not been successfully uncoupled, distorting the measured optical signal. (For interpretation of the references to colour in this figure legend, the reader is referred to the web version of this article).

Optically recorded voltage signals often exhibit low and variable signal to noise ratios (SNR), due to short exposure times and heterogeneous loading of the dyes which exhibit small fractional fluorescent changes. Low SNR, coupled with large datasets constituting thousands of image frames, means that substantial data processing is required before data can be analysed, Fig. 2A. Complexity of processing and analysis of this data has therefore previously acted as a barrier to uptake of the optical mapping technology. However, recent developments have seen the release and publication of open-source software dedicated to the analysis of optical mapping data (Laughner et al., 2012; O'Shea et al., 2019b), including panoramic analysis (Gloschat et al., 2018).

### 3.3 Panoramic optical mapping

Optical mapping is predominantly performed with a single camera. Thus, the curvature and surface topology of the 3D cardiac structures imaged is visible only as a 2D projection. This may introduce artefacts to the activation and repolarisation times measured. Furthermore, viewing only one part of the heart is clearly inferior to multi-angle panoramic imaging and can lead to researchers missing important phenomena outside the field of view, Fig. 2B. Investigators have hence introduced methods to undertake panoramic optical mapping with projection of the optical data onto the 3D surface. Originally, mirrors were utilised to achieve panoramic mapping in rabbit hearts (Bray et al., 2000). Subsequently panoramic optical mapping was extended to larger animal hearts using multi-camera solutions (Kay et al., 2004). Recently, panoramic optical mapping has benefited from the open source availability of both 3D printable hardware and analysis software, (Gloschat et al., 2018). Nevertheless, whether panoramic optical mapping completely overcomes the curvature problems remains to be investigated.

### 3.4 Electromechanical mapping

Freely beating cardiac tissue has presented a significant challenge to optical mapping. As tissue moves, the area being imaged by a specific pixel will change, distorting the recorded time course of that pixel, Fig. 2C. To overcome this challenge, excitation-contraction uncouplers are employed. These molecules (*e.g.* Blebbistatin) prevent cardiomyocyte contraction but not electrical excitation. They hence allow continuous recording of undistorted signals at each pixel location, but in mechanically uncoupled hearts.

The uncoupled heart has a reduced energy and oxygen demand (Garrott et al., 2017), lacks bidirectional interaction between electrical and mechanical activity, and cannot be studied *in vivo* without approaches such as cardiopulmonary bypass (Lee et al., 2012). Some studies suggest that the uncoupling agent Blebbistatin exerts additional direct side effects on cardiac electrophysiology (Brack et al., 2013). However, there are conflicting reports on the direct and indirect side effects of Blebbistatin on cardiac electrophysiology (Fedorov et al., 2007), and the reader is directed to (Kappadan et al., 2020) for a thorough discussion on this subject.

For these reasons, technical and computational approaches have been applied to realise optical mapping in freely beating preparations. Excitation or emission ratiometry allows recording of two simultaneous signals from the heart, both similarly corrupted by motion artefacts but differentially responsive to voltage (or calcium concentration). This enables mitigation of motion artefacts (Knisley et al., 2000; Bachtel et al., 2011). Ratiometry alone however is often not sufficient to fully remove motion artefacts owing to heterogeneous illumination and/or dye loading. Therefore, ratiometry has been combined with marker tracking procedures to both remove motion artefacts, and map mechanical deformations simultaneously with optically recorded transmembrane voltage (Zhang et al., 2016; Garrott et al., 2017). Furthermore, methodologies such as optical flow have been utilised to track motion without the requirement of fiducial markers (Kappadan et al., 2020). These methods still operate under certain constraints, for example homogenous illumination and sufficient regional contrasts, to be successfully applied (Christoph and Luther, 2018). Whilst these approaches are currently not widely adopted, they demonstrate how the reliance of optical mapping on un-physiological motion uncoupling can be overcome.

### 3.5 *In vivo* optical mapping

Several of the challenges already discussed, (*e.g.* use of mechanical uncouplers, dye toxicity, scattering and absorbance of optical signals by biological chromophores) limit optical mapping to primarily ex vivo applications. Achieving optical mapping in an *in vivo* setting, with a freely beating blood perfused heart with intact circulation and nervous system, would elevate the translatability of optical mapping and likely lead to novel insights into arrhythmogenic drivers. Early attempts showing the potential for *in vivo* optical mapping physically attached optical fibres to the heart to suppress motion artefacts with limited success (Dillon et al., 1998), while cardiopulmonary bypass has been employed to permit the use of mechanical uncouplers in living anaesthetised animals (Lee et al., 2012).

More recently, developments such as excitation ratiometry of high sensitivity red-shifted fluorescent dyes have been exploited to obtain optical action potentials from beating porcine hearts *in vivo* in an open chest model (Lee et al., 2019). Using an EMCCD camera, electrical conduction could be mapped at high resolution during epicardial pacing and ventricular fibrillation. Limitations remain nonetheless, with persistent motion artefacts during pacing precluding effective mapping of repolarisation dynamics during far-field imaging. However, by imaging instead with an optical fibre array pressed against the epicardial surface, activation and repolarisation could be effectively mapped, although at a low resolution of 4 × 4 pixels (Lee et al., 2019). Further advances, including the implementation of sophisticated motion tracking techniques (Christoph and Luther, 2018; Kappadan et al., 2020) and higher resolution optical fibre arrays will potentially fuel a new era of *in vivo* optical mapping experiments.

### 3.6 Integrated cardiac optogenetics and optical mapping

While optical mapping focuses on the use of light to interrogate cardiac electrophysiology, cardiac optogenetics allows light to be used to activate, supress or modulate electrical behaviour. This is achieved by the expression of opsins, light sensitive ion channels and pumps, in cardiac preparations. Combination of optical mapping with optogenetics in sophisticated setups has seen the advent of all-optical electrophysiology. These setups combine the high-resolution study of optical mapping with precise, immediate actuation of cardiac tissue by opsin activation (O'Shea et al., 2019a). Thus, all-optical electrophysiology can be used for innovative experimentation, such as ‘real-time simulations’ in cardiac cell monolayers (Burton et al., 2015) and tissue (Scardigli et al., 2018).

## 4 Conclusion

Optical mapping has provided valuable insights into cardiac electrophysiology in both health and disease. Using fluorescent indicators rather than electrodes, it is possible to directly record cellular electrical activity at spatial resolutions not possible with electrode techniques. Optically recording the electrical activity of the heart also provides unique challenges such as signal attenuation due to interaction of light with cardiac tissue, motion artefacts, low and heterogenous signal quality and 2D distortion of complex 3D geometries. Recent advances however are overcoming these challenges, including development of novel fluorescent dyes, panoramic imaging systems and technical and computational approaches to allow recording from freely beating hearts. Taken together, these advances are further enhancing the unique role optical mapping plays in pre-clinical cardiac research, including moving the technique towards direct *in vivo* optical mapping of cardiac electrophysiology.

## Funding information

This work was funded by the EPSRC studentship (Sci-Phy-4-Health Centre for Doctoral TrainingL016346) to DP, KR and LF. Wellcome Trust Seed Award Grant (109604/Z/15/Z) to DP. British Heart Foundation Grants (PG/17/55/33087, RG/17/15/33106) to DP, PG/17/30/32961 to AH, Accelerator AwardAA/18/2/34218 to Institute of Cardiovascular Sciences (LF).
